# Expanding the spectrum of IPEX: from new clinical findings to novel treatments

**DOI:** 10.1097/ACI.0000000000001033

**Published:** 2024-10-11

**Authors:** Marta Voarino, Filippo Consonni, Eleonora Gambineri

**Affiliations:** a Department of Health Sciences; b Department of Experimental and Clinical Biomedical Sciences “Mario Serio”, University of Florence; c Division of Pediatric Oncology/Hematology, Meyer Children's Hospital IRCCS; d Department of Neurosciences, Psychology, Drug Research and Child Health (NEUROFARBA), University of Florence, Florence, Italy

**Keywords:** FOXP3, immune dysregulation, inborn errors of immunity, immune dysregulation, polyendocrinopathy, enteropathy, X-linked, regulatory T cells

## Abstract

**Purpose of review:**

This review aims to provide an overview of recent research findings regarding immune dysregulation, polyendocrinopathy, enteropathy, X-linked (IPEX) syndrome, focusing on clinical and immunological novelties, as well as emerging treatment strategies, based on the published literature of the last few years.

**Recent findings:**

While it is well known that IPEX can present with a wide range of atypical clinical manifestations, new and unique phenotypes continue to emerge, making it essential to maintain a high level of clinical suspicion both at the time of diagnosis and during follow-up. This unpredictability in clinical presentation is further compounded by the lack of a clear genotype-phenotype correlation. A valuable tool for monitoring comes from recent discoveries regarding the epigenetic signature of Tregs, which, by correlating with disease severity, could prove to be a useful biomarker for diagnosis and ongoing management. The use of biological agents is emerging as an alternative to traditional immunosuppression. Additionally, ongoing studies are exploring the feasibility of gene therapy through the introduction of the wild-type *FOXP3* into peripheral CD4^+^ T cells.

**Summary:**

Further research is needed to fully understand the variable clinical presentations of IPEX and optimize tailored therapies, ensuring better management and outcomes for affected individuals.

## INTRODUCTION

Immune dysregulation, polyendocrinopathy, enteropathy, X-linked (IPEX) syndrome is a rare monogenic disease caused by mutations in the Forkhead Box Protein 3 (*FOXP3*) gene. *FOXP3* encodes a transcription factor fundamental for the function of regulatory T cells (Tregs). Mutations of this gene alter T cells homeostasis and peripheral tolerance, leading to overwhelming immune dysregulation and autoimmunity, often fatal in the first year of life [1]. The classic clinical presentation of IPEX is characterized by the triad of early-onset intractable diarrhea, type 1 diabetes (T1D), and eczema. Being the prototypical monogenic autoimmune disease, many patients develop additional autoimmune manifestations such as autoimmune hepatitis, thyroiditis, autoimmune hemolytic anemia, and nephropathy [2]. Moreover, there is growing evidence in the literature of atypical presentations characterized by late-onset, milder clinical phenotypes, or rare autoimmune manifestations, including single-organ involvement [2]. Despite the heterogeneous clinical manifestations, early diagnosis is pivotal for establishing optimal care. Currently, the mainstays of treatment are immunosuppression and hematopoietic stem cell transplantation (HSCT), which is the definitive curative approach [3]. Recent advances in gene therapy hold promise for making this a feasible and safe curative option for IPEX patients. Through this review, we aim to shed light on recent findings, including new clinical and immunological insights as well as innovative treatment approaches for this rare inborn error of immunity (IEI). 

**Box 1:**
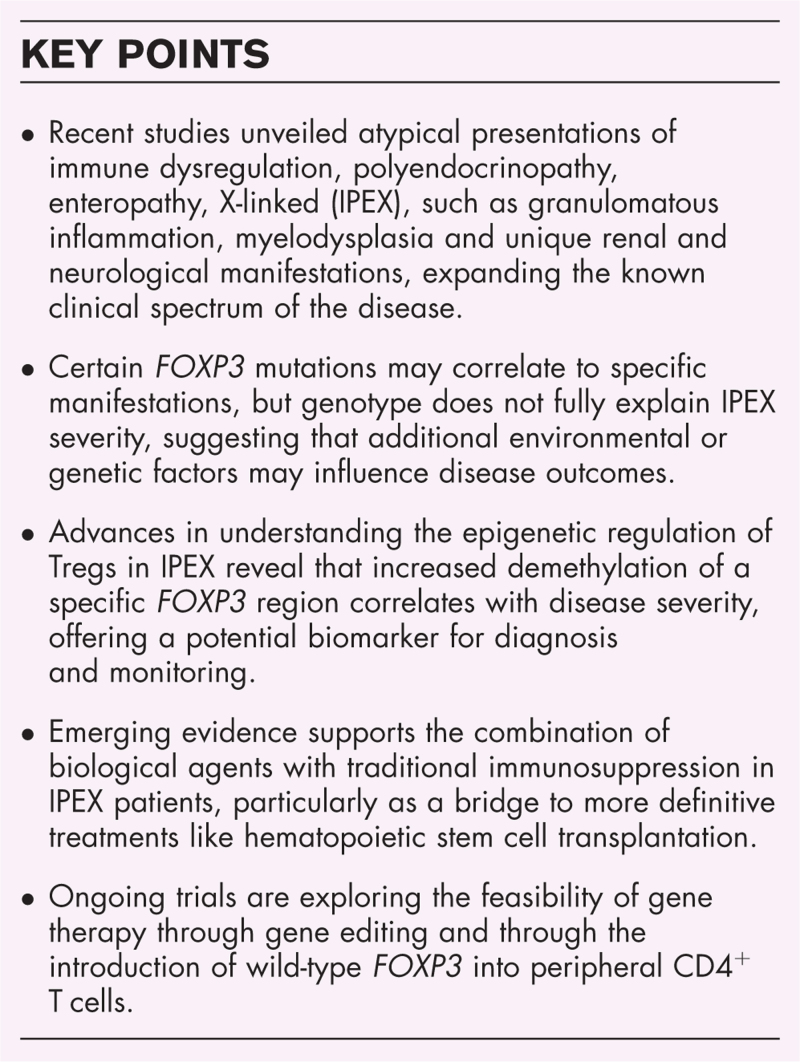
no caption available

## NOVEL CLINICAL FINDINGS

Considering the inherently polymorphic nature of the clinical presentation of IPEX, it is important to maintain a high clinical suspicion in cases of autoimmunity or immune dysregulation patterns presenting with red flags such as early onset, multiorgan involvement, or poor response to classical treatment, even when symptoms extend beyond the classical presentation of IPEX. For example, Duztas *et al.* recently described a patient affected by IPEX who presented with neonatal onset of inflammatory bowel disease associated with granulomas in the gastrointestinal tract and lung [4]. To our knowledge, a granulomatous response has not been previously described as part of IPEX histopathology [5], nevertheless it could be interpreted as part of a dysregulated immune response, highlighting the extremely variable clinical and histopathological nature of this condition.

**FIGURE 1:**
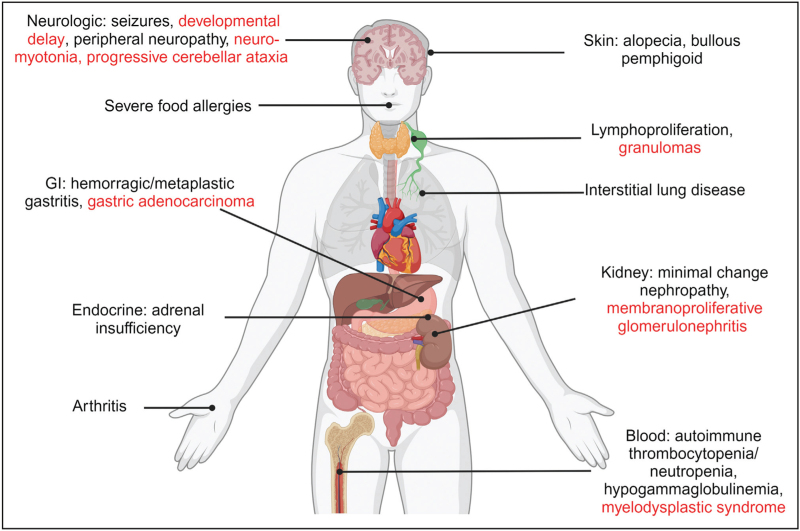
Atypical clinical manifestations of IPEX sorted by organ involvement. Captions in black correspond to known atypical manifestations, while those in red correspond to recently described atypical manifestations.

In the following sections, we have gathered the most significant reports of atypical manifestations of IPEX described in recent years, categorized by the organs involved (Fig. 1, Table 1).

**Table 1:** Clinical manifestations of IPEX sorted by organ involvement

Organ involvement	Classical triad	Other typical manifestations	Known atypical manifestations	Newly reported atypical manifestations
Skin	Eczema	Other dermatitis	Alopecia, bollous pemphigoid	
Central/peripheral nervous system			Seizures, peripheral neuropathy	Developmental delay, neuro-myotonia, progressive cerebellar ataxia
Pulmonary			Interstitial lung disease	
Gastrointestinal	Chronic enteropathy	Failure to thrive, autoimmune hepatitis	Hemorragic/metaplastic gastritis	Gastric adenocarcinoma
Endocrine	Type I diabetes mellitus	Thyroiditis	Adrenal insufficiency	
Articular			Arthritis	
Urogenital		Interstitial/membranous nephropathy	Minimal change nephropathy	Membranoproliferative glomerulonephritis
Immune system		Severe/recurrent infections	Lymphoproliferation, severe food allergies, hypogammaglobulinemia	Granulomas, anti-INF antibodies (?)
Hematological		Autoimmune hemolytic anemia	Autoimmune thrombocytopenia, autoimmune neutropenia	Myelodysplastic syndrome

The columns differentiate between the classic triad, typical manifestations, atypical manifestations, and recently described atypical manifestations.

### Renal involvement

Some of the typical clinical manifestations of IPEX can be influenced by the chronic immunosuppression that many of these patients undergo, making it challenging to differentiate between the side effects of therapy and the disease's inherent manifestations, especially given the polymorphic nature of the condition. This difficulty is particularly evident in pulmonary and renal involvement, and increased susceptibility to infections.

Regarding renal involvement, several authors have described patients with early kidney disease that precedes the initiation of immunosuppressive therapy, supporting an autoimmune pathogenesis. Moreover, a systematic review conducted on neonates with genetically-confirmed IPEX by Hou *et al.* revealed that 23% (13/55) of patients presented with kidney involvement, with nephrotic syndrome being the most commonly represented manifestation [6^▪^].

Specifically, the most frequently described nephropathy in patients with IPEX is membranous nephropathy (MN) [7,8^▪^], an autoimmune disease associated with nephrotic syndrome. In this case, damage to renal podocytes is mediated by the presence of autoantibodies against M-type phospholipase A2 receptor (PLA2R). However, some cases of IPEX patients were described without anti-PLA2R antibodies [7,9]. On the other hand, Miller *et al.* described a patient with double positivity on immunohistochemistry to PLA2R and Semaphorin-3b (SEMA3b) autoantigens on renal biopsy, highlighting the fact that there may be other target antigens involved in the pathophysiology of IPEX-related membranous nephropathy [10,11].

Additionally, cases of tubulointerstitial nephritis, interstitial nephritis, minimal change disease (MCD) and membranoproliferative glomerulonephritis have also been reported [7,8^▪^]. A multicenter study conducted on patients affected by podocytopathies revealed that antinephrin antibodies may play a pathophysiological role in MCD [12]. Applying this concept to IPEX, this finding supports the hypothesis that MCD in IPEX patients may have an autoimmune basis. Further research is needed to investigate the presence of antinephrin antibodies in IPEX patients with kidney involvement, particularly in those with active MCD.

### Susceptibility to infections

Susceptibility to infections in IPEX is mainly related to chronic immunosuppression and disease-related organ damage. Nevertheless, the relatively high incidence of infections caused by *Staphylococci*, *Cytomegalovirus*, and *Candida*[13] raises the question of a pathogenic role for anticytokine antibodies. Indeed, autoantibodies against interferon-α (IFN-α) with *in vitro* blocking capacity have been detected in the sera of IPEX patients, albeit at lower titers compared to patients with defects affecting the central tolerance mechanism, such as autoimmune polyendocrine syndrome type I [14]. While the presence of antibodies against type I interferons (IFNs) has been linked to acquired immunodeficiency and increased susceptibility to certain viral infections, including SARS-CoV-2 [15], a direct clinical correlate has not been observed among IPEX patients. To our knowledge, severe COVID-19 in IPEX patients has not been described in the literature, although an ongoing international study may provide further insights on this topic. Recently, an interesting case of IPEX presenting with severe herpes simplex virus (HSV) encephalitis in a previously-healthy toddler was described [16]. Unfortunately, it was not possible to measure autoantibodies against type I IFNs in this case.

### Neurological manifestations

Previous studies highlighted that IPEX patients may display various neurological abnormalities ranging from seizures and developmental delay to peripheral neuropathy and muscles disorders [3,13]. Moreover, a recent case study described the presence of antivoltage-gated potassium channel (VGKC) antibodies associated with neuromyotonia that resolved following HSCT in three patients affected by IPEX [17]. These autoantibodies can alter neuronal excitability and have been associated with various clinical manifestations of the central, peripheral and autonomic nervous system, including isolated epilepsy and limbic encephalitis. Therefore, the presence of these autoantibodies should be excluded in IPEX patients presenting with neurological syndromes. Nevertheless, it is sometimes not possible to identify the underlying cause of the clinical neurological manifestation, as in the case recently described by Rim *et al.* of a young adult affected by IPEX presenting with progressive cerebellar ataxia of unknown origin [18].

### Onco-hematological manifestations

While blood cytopenia is a typical clinical manifestation of IPEX – as well as of other IEI [19] – Toyama *et al.* described the first and only reported case of IPEX presenting with myelodysplastic syndrome [20]. The clinical picture was further complicated by the presence of autoantibodies against red blood cells and platelets, as well as clinical response to immunosuppressive therapy. While these findings may appear contradictory at first, they underscore both the influence of immune dysregulation on the pathogenesis of myelodysplasia [21] and the pleomorphic origin of cytopenia in pathologies affecting Tregs [22]. Indeed, some IEI, such as CTLA-4 deficiency, present with cytopenias associated with both bone marrow failure and autoimmunity, making them “interface disorders”. While this has not been demonstrated in IPEX patients, it seems natural that the loss of Treg function alongside the chronic inflammatory environment may significantly influence bone marrow function.

FOXP3 is expressed in epithelial tissue of breast, lung and prostate where it acts as a tumor-suppressor gene, as proven by the presence of genetic alteration in *FOXP3* in human breast cancer samples [23]. Even though IPEX has not been linked to susceptibility to tumors, the first case of a solid tumor in a 14-year-old boy affected by gastric adenocarcinoma was recently described [24]. While the causative link between IPEX and oncogenesis remains elusive, alterations in T cells homeostasis and reduced expression of FOXP3 in the epithelial cells may have played a role in the development of this patient's cancer.

## NEW IMMUNOLOGICAL FINDINGS

### IPEX immunogenetics

A clear genotype–phenotype correlation has never been established in IPEX [3]. Clinical findings in siblings with completely different disease severity suggest that other immunological or environmental factors could contribute to the development of the autoimmune phenotype [25^▪▪^]. A recent study confirms this hypothesis, reproducing human *FOXP3* mutations in mice *Foxp3* via CRISPR/Cas9 [26]. Only the R337Q mutation (Forkhead domain) spontaneously induced multiorgan autoimmunity, while variants in any other *Foxp3* domain determined clinical findings only after certain “immunological challenges” or if co-expressed with certain autoimmune-prone alleles. Nevertheless, we previously showed that certain *FOXP3* mutations may be associated to peculiar clinical features [2], and recent observations of a correlation between the c.1222 G>A variant and muscular manifestations furtherly confirm this aspect [8^▪^].

We earlier demonstrated the inconsistent correlation between the clinical phenotype of IPEX and FOXP3 expression [27]. A recent study provides further evidence to this point, showing that in IPEX patients, the frequency of Tregs expressing FOXP3 was only slightly inferior to that of healthy individuals, though its intensity was often lower [25^▪▪^]. Similarly, other immunological biomarkers were similar between the two groups, while IPEX patients showed increased memory CD4^+^ T cells, increased eosinophils, and immunoglobulin E (IgE) levels, as seen in other immunodeficiencies with immune dysregulation [28–30].

### Role of *FOXP3* exon 2

Another topic to address is the role of the two main isoforms of human FOXP3: the full-length version (FOXP3FL) and the one lacking exon 2 (FOXP3Δ2). Since mice express only the former (Foxp3FL), the role of FOXP3Δ2 in humans has never been clearly established, aside from evidence showing that its presence, in the absence of FOXP3FL, does not prevent the development of IPEX but mitigates its clinical phenotype [31]. In a recent study, Du *et al.* generated a Foxp3Δ2 murine model, showing that the absence of exon 2 expression did not hinder the generation and suppressive function of Tregs. However, Foxp3Δ2 mice exhibited unstable Tregs and later developed features of systemic, lupus-like autoimmunity, characterized by the production of antinuclear and antidsDNA antibodies, along with excessive T follicular helper cells and germinal center B cell responses [32^▪^]. Therefore, while *FOXP3*'s exon 2 may not be crucial for Treg generation, it is essential for ensuring Treg stability and maintaining immune homeostasis.

### Novel implications of Treg epigenetic signature in IPEX

Treg-cell-specific demethylation region (TSDR) is a specific CpG island in the *FOXP3*'s conserved noncoding sequence 2 (CNS2). It acts a signature of Treg cells, independently of FOXP3 expression. In IPEX patients, the number of T cells with fully demethylated *FOXP3* TSDR is typically higher in peripheral blood [8^▪^]. Recent studies used TSDR to track Treg cells in IPEX and, in a clinical setting, attempted to correlate TSDR levels with the severity of the disease [25^▪▪^,^33^^▪▪^,^34^]. Borna *et al.* combined TSDR analysis with single-cell multiomics and bulk T-cell receptor (TCR) sequencing, revealing that certain Tregs in IPEX patients lose their characteristic markers (i.e., CD25 and FOXP3) to acquire effector T cell (Teff) features, including the attainment of Th2-like functions. This metamorphosis may directly cause the immune dysregulation of IPEX, but it can be reversed through HSCT, which introduces FOXP3-wild type Tregs [33^▪▪^]. In the clinics, assessment of TSDR is a useful biomarker of the degree of immune dysregulation in IPEX, since increased demethylation correlates with a typical IPEX phenotype [25^▪▪^]. Moreover, TSDR analysis could support the validation of variants of unknown significance in *FOXP3*, accelerating diagnosis and treatment of IPEX [34]. These studies also provide insight into the Th2-like characteristics of IPEX, such as eosinophilia and elevated IgE levels. These are caused by increased Th2-like proinflammatory cytokines [25^▪▪^], most likely produced by unstable and dysregulated Tregs that transform into Th2-like Teff cells [33^▪▪^].

## NEW TREATMENTS

Being classical IPEX fatal within the first year of life, prompt treatment is pivotal. Apart from supportive therapies, the first line of treatment is usually based on pharmacological immune suppression, achieved through steroids, calcineurin inhibitors or rapamycin, according to the clinical picture [35]. In particular, rapamycin was proven to increase the suppressive function of Treg cells [36], acting not only as a symptomatic drug but also improving the immunological profile in IPEX patients. Therefore, even in consideration of its rather favorable safety profile, it has been increasingly used also in monotherapy in patients with mild phenotypes. Nevertheless, chronic immunosuppression leads to significant side effects such as increased infections and ultimately does not prevent disease progression and development of complications [3].

Curative therapy able to correct the genetic defect is achieved through allogenic HSCT. However, it is important to consider the benefit-risk ratio for each patient when determining the timing of the transplant. Indeed, on one hand, HSCT bears procedure-related mortality, long-term complications and risk of disease recurrence, which can reduce overall survival to 60% [8^▪^]. On the other, delaying the transplant increases the risk of the patient accumulating irreversible end-organ damage, which can worsen the prognosis of the transplant itself. These considerations must be taken into account in cases where the patient is clinically well controlled with pharmacological immune suppression.

In this context, new approaches with biological agents such as Abatacept, Infliximab and Dupilumab are emerging as an alternative in patients with disease that is resistant to multiple lines of immune suppressive drugs (Table 2) [37,38]. Interestingly, a new study conducted by Gerbaux *et al.* on *Foxp3* knock-out mice revealed a better clinical and immunological outcome in mice treated with Abatacept, a CTLA4 fusion protein inhibiting T cell activation, compared to those treated with rapamycin [39^▪^]. These biological agents may even be considered as a bridge to transplant, in order to control the disease and prevent end organ-damage while waiting for definite treatment with HSCT.

**Table 2:** Novel target therapy options and recent evidence of efficacy in IPEX

Target treatment	Molecular mechanism	Recent evidence of efficacy in IPEX	References
Abatacept	Anti CD80/CD86 (CTLA4 analog)	Beneficial in *Foxp3* knockout mice: better clinical and immunological outcome as well as better engraftment following HSCT	Gerbaux *et al.*[39^▪^]
Dupilumab	Anti IL-4/IL-13 (binding IL-4Rα subunit)	Case report of clinical control and steroid-tapering in a patient resistant to different lines of immune suppression	Caruso *et al.*[38]
Infliximab	Anti TNFα	Case report of clinical control and improved immunological profile in a patient resistant to different lines of immune suppression	Boschetti *et al.*[37]
Rapamycin	mTOR kinase inhibitor	Partially restored Treg suppressive function via FOXP3-independent mechanism	Passerini *et al.*[36]

CTLA4, cytotoxic T-lymphocyte Antigen 4; Foxp3, forkhead box P3; HSCT, hematopoietic stem cells transplantation; IL, interleukin; IL-4Rα, interleukin 4 receptor alpha; mTOR, mammalian target of rapamycin; TNFα, tumor necrosis factor alpha.

In recent years, advancements in gene transfer and gene editing technologies broadened the horizons of gene therapy. Given that IPEX is a monogenic condition, it represents an ideal candidate for such therapeutic approaches. Studies conducted on mice have demonstrated that the genetic makeup of autologous hematopoietic stem cells (HSCs) can be corrected using different methods, such as lentiviral vector insertion of the corrected *FOXP3*[40] or clustered regularly interspaced short palindromic repeat (CRISPR)-associated protein 9 (Cas9)-based gene correction [41]. Following engraftment, these HSCs can undergo multilinear and terminal differentiation, ultimately leading to the production of Tregs expressing the corrected *FOXP3* gene. Even though the results obtained in terms of FOXP3 expression or frequency of Tregs are still suboptimal, these findings open up on exiting new prospects for the treatment of IPEX.

Moreover, since the pathophysiology of IPEX is entirely based on the loss of function of *FOXP3* in Tregs, theoretically it would be possible to cure this condition by introducing only peripheral CD4^+^ T cells carrying the corrected *FOXP3*, instead of correcting the entire hematopoietic stem cells pool. However, a key uncertainty of this approach will be the durability and stability of the infused Tregs *in vivo*. Currently, there is an ongoing phase I clinical trial (NCT05241444) testing feasibility and safety of infusion of autologous CD4^+^ T cells that have undergone lentiviral-mediated gene transfer of *FOXP3*.

## CONCLUSION

Since new and unique IPEX clinical manifestations continue to emerge, it is fundamental to keep a high level of clinical suspicion during diagnosis and follow-up. Further research is needed to fully understand the variable clinical presentation of IPEX and optimize tailored therapies, ensuring better management and outcomes for affected individuals.

## Acknowledgements

*This study was supported in part by funds from the “Current Research Annual Funding” of the Italian Ministry of Health.*

*The authors declare that the research was conducted in the absence of any commercial or financial relationships that could be construed as a potential conflict of interest.*

*Figure 1* *was created with Biorender.com and was exported under a paid subscription.*

### Financial support and sponsorship

*None.*

### Conflicts of interest

*There are no conflicts of interest.*

## REFERENCES AND RECOMMENDED READING

Papers of particular interest, published within the annual period of review, have been highlighted as:

▪ of special interest
▪▪ of outstanding interest

## References

[1] Bacchetta R Barzaghi F Roncarolo M-G. From IPEX syndrome to FOXP3 mutation: a lesson on immune dysregulation. Ann N Y Acad Sci 2018; 1417:5–22. 26918796 10.1111/nyas.13011

[2] Consonni F Ciullini Mannurita S Gambineri E. Atypical presentations of IPEX: expect the unexpected. Front Pediatr 2021; 9:643094. 33614561 10.3389/fped.2021.643094 PMC7892580

[3] Barzaghi F Hernandez LCA Neven B. Long-term follow-up of IPEX syndrome patients after different therapeutic strategies: an international multicenter retrospective study. J Allergy Clin Immunol 2018; 141:1036–1049. e5. 29241729 10.1016/j.jaci.2017.10.041 PMC6050203

[4] Duztas DT Al-Shadfan L Ozturk H. New findings of immunodysregulation, polyendocrinopathy, and enteropathy X-linked syndrome (IPEX); granulomas in lung and duodenum. Pediatr Dev Pathol 2021; 24:252–257. 33683986 10.1177/1093526621998868

[5] Patey-Mariaud de Serre N Canioni D Ganousse S. Digestive histopathological presentation of IPEX syndrome. Mod Pathol 2009; 22:95–102. 18820676 10.1038/modpathol.2008.161

[6▪] Hou A-N Wang Y Pan Y-Q. A case report of IPEX syndrome with neonatal diabetes mellitus and congenital hypothyroidism as the initial presentation, and a systematic review of neonatal IPEX. J Clin Immunol 2023; 43:979–988. 36867340 10.1007/s10875-023-01456-0

[7] Sheikine Y Woda CB Lee PY. Renal involvement in the immunodysregulation, polyendocrinopathy, enteropathy, X-linked (IPEX) disorder. Pediatr Nephrol 2015; 30:1197–1202. 25911531 10.1007/s00467-015-3102-x

[8▪] Bacchetta R Roncarolo MG. IPEX syndrome from diagnosis to cure, learning along the way. J Allergy Clin Immunol 2024; 153:595–605. 38040040 10.1016/j.jaci.2023.11.021

[9] Tan L An Y Yang Q. A novel FOXP3 mutation in a Chinese child with IPEX-associated membranous nephropathy. Mol Genet genomic Med 2022; 10:e1945. 35434975 10.1002/mgg3.1945 PMC9184667

[10] Miller P Lei L Charu V. Clinicopathologic features of nonlupus membranous nephropathy in a pediatric population. Pediatr Nephrol 2022; 37:3127–3137. 35333973 10.1007/s00467-022-05503-7

[11] Sethi S Debiec H Madden B. Semaphorin 3B-associated membranous nephropathy is a distinct type of disease predominantly present in pediatric patients. Kidney Int 2020; 98:1253–1264. 32534052 10.1016/j.kint.2020.05.030

[12] Hengel FE Dehde S Lasse M. Autoantibodies targeting nephrin in podocytopathies. N Engl J Med 2024; 391:422–433. 38804512 10.1056/NEJMoa2314471

[13] Gambineri E Mannurita SC Hagin D. Patients with the phenotype of immune dysregulation, polyendocrinopathy, enteropathy, X-linked (IPEX) syndrome. Front Immunol 2018; 9:2411. 30443250 10.3389/fimmu.2018.02411 PMC6223101

[14] Rosenberg JM Maccari ME Barzaghi F. Neutralizing anti-cytokine autoantibodies against interferon-α in immunodysregulation polyendocrinopathy enteropathy X-linked. Front Immunol 2018; 9:544. 29651287 10.3389/fimmu.2018.00544 PMC5885158

[15] Bastard P Rosen LB Zhang Q. Autoantibodies against type I IFNs in patients with life-threatening COVID-19. Science 2020; 370:eabd4585. 32972996 10.1126/science.abd4585 PMC7857397

[16] Rossini L Bonardi CM Bresolin S. Severe herpes simplex encephalitis: an unusual presentation of IPEX. J Clin Immunol 2024; 44:100. 38625673 10.1007/s10875-024-01702-z

[17] Moseley N King J Van Dort B. Antivoltage-gated potassium channel (VGKC) antibodies and acquired neuromyotonia in patients with immune dysregulation, polyendocrinopathy, enteropathy X-lined (IPEX) syndrome. J Clin Immunol 2021; 41:1972–1974. 34478044 10.1007/s10875-021-01128-x

[18] Rim J Byler M Soldatos A. Opinion and special articles: cerebellar ataxia and liver failure complicating IPEX syndrome. Neurology 2021; 96:e956–e959. 33168705 10.1212/WNL.0000000000011195 PMC8105902

[19] Schiavo E Martini B Attardi E. Autoimmune cytopenias and dysregulated immunophenotype act as warning signs of inborn errors of immunity: results from a prospective study. Front Immunol 2021; 12:790455. 35058929 10.3389/fimmu.2021.790455 PMC8765341

[20] Toyama D Hoshino A Inoue K. Myelodysplastic syndrome in a patient with IPEX syndrome. J Clin Immunol 2021; 41:1683–1685. 34251574 10.1007/s10875-021-01092-6

[21] Alfinito F Sica M Luciano L. Immune dysregulation and dyserythropoiesis in the myelodysplastic syndromes. Br J Haematol 2010; 148:90–98. 19793254 10.1111/j.1365-2141.2009.07921.x

[22] Bleesing J. Gain-of-function defects in toll-like receptor 8 shed light on the interface between immune system and bone marrow failure disorders. Front Immunol 2022; 13:935321. 36119097 10.3389/fimmu.2022.935321 PMC9479092

[23] Liu R Li S Yang W-H Wang L. IPEX syndrome, FOXP3 and cancer. J Syndr 2013; 1:7. 25844400 10.13188/2380-6036.1000001 PMC4383293

[24] Steffin D Bhar S Fishman DS. Gastric adenocarcinoma in the setting of IPEX syndrome. Case Rep Immunol 2021; 2021:9967198. 10.1155/2021/9967198 PMC8257369 34258086

[25▪▪] Narula M Lakshmanan U Borna S. Epigenetic and immunological indicators of IPEX disease in subjects with FOXP3 gene mutation. J Allergy Clin Immunol 2023; 151:233–246. e10. 36152823 10.1016/j.jaci.2022.09.013

[26] Leon J Chowdhary K Zhang W. Mutations from patients with IPEX ported to mice reveal different patterns of FoxP3 and Treg dysfunction. Cell Rep 2023; 42:113018. 37605532 10.1016/j.celrep.2023.113018 PMC10565790

[27] Gambineri E Perroni L Passerini L. Clinical and molecular profile of a new series of patients with immune dysregulation, polyendocrinopathy, enteropathy, X-linked syndrome: inconsistent correlation between forkhead box protein 3 expression and disease severity. J Allergy Clin Immunol 2008; 122:1105–1113. 18951619 10.1016/j.jaci.2008.09.027

[28] James AE Abdalgani M Khoury P. TH2-driven manifestations of inborn errors of immunity. J Allergy Clin Immunol 2024; 154:245–254. 38761995 10.1016/j.jaci.2024.05.007 PMC12295673

[29] Pontone M Giovannini M Filippeschi C. Biological treatments for pediatric Netherton syndrome. Front Pediatr 2022; 10:1074243. 36619513 10.3389/fped.2022.1074243 PMC9822572

[30] Levy R. Human CARMIL2 deficiency underlies a broader immunological and clinical phenotype than CD28 deficiency. J Exp Med 2023; 220:e20220275. 36515678 10.1084/jem.20220275 PMC9754768

[31] Frith K Joly A Ma CS. The FOXP3Δ2 isoform supports Treg cell development and protects against severe IPEX syndrome. J Allergy Clin Immunol 2019; 144:317–320. e8. 30904640 10.1016/j.jaci.2019.03.003

[32▪] Du J Wang Q Yang S. FOXP3 exon 2 controls Treg stability and autoimmunity. Sci Immunol 2022; 7:1–15. 10.1126/sciimmunol.abo5407 PMC9333337 35749515

[33▪▪] Borna Š Lee E Nideffer J. Identification of unstable regulatory and autoreactive effector T cells that are expanded in patients with FOXP3 mutations. Sci Transl Med 2023; 15: doi: 10.1126/scitranslmed.adg6822. [Epub ahead of print]. 10.1126/scitranslmed.adg6822 PMC11070150 38117899

[34] Wyatt RC Olek S De Franco E. FOXP3 TSDR measurement could assist variant classification and diagnosis of IPEX syndrome. J Clin Immunol 2023; 43:662–669. 36600150 10.1007/s10875-022-01428-w PMC9957900

[35] Consonni F Favre C Gambineri E. IL-2 signaling axis defects: how many faces? Front Pediatr 2021; 9:669298. 34277517 10.3389/fped.2021.669298 PMC8282996

[36] Passerini L Barzaghi F Curto R. Treatment with rapamycin can restore regulatory T-cell function in IPEX patients. J Allergy Clin Immunol 2020; 145:1262–1271. e13. 31874182 10.1016/j.jaci.2019.11.043

[37] Boschetti G Sarfati M Fabien N. Infliximab induces clinical resolution of sacroiliitis that coincides with increased circulating FOXP3+ T cells in a patient with IPEX syndrome. Jt Bone Spine 2020; 87:483–486. 10.1016/j.jbspin.2020.04.013 32438064

[38] Caruso C Laterza L Settanni CR. Case report: dupilumab treatment improved type 2 disorders in a patient with IPEX syndrome diagnosis. Front Immunol 2023; 13:995304. 36713411 10.3389/fimmu.2022.995304 PMC9875030

[39▪] Gerbaux M Roos E Willemsen M. CTLA4-Ig effectively controls clinical deterioration and immune condition in a murine model of Foxp3 deficiency. J Clin Immunol 2023; 43:1393–1402. 37156988 10.1007/s10875-023-01462-2 PMC10354160

[40] Masiuk KE Laborada J Roncarolo MG. Short article lentiviral gene therapy in HSCs restores lineage-specific Foxp3 expression and suppresses autoimmunity in a mouse model of IPEX syndrome short article lentiviral gene therapy in HSCs restores lineage-specific foxp3 expression and suppresses. Stem Cell 2019; 24:309–317. e7. 10.1016/j.stem.2018.12.003 30639036

[41] Goodwin M Lee E Lakshmanan U. CRISPR-based gene editing enables FOXP3 gene repair in IPEX patient cells. Sci Adv 2020; 6:eaaz0571. 32494707 10.1126/sciadv.aaz0571 PMC7202871

